# Identification of genome compositions in allopolyploid species of the genus Elymus (Poaceae: Triticeae) in the Asian part of Russia by CAPS analysis

**DOI:** 10.18699/VJ20.606

**Published:** 2020-03

**Authors:** A.V. Agafonov, E.V. Shabanova (Kobozeva), S.V. Asbaganov, A.V. Mglinets, V.S. Bogdanova

**Affiliations:** Central Siberian Botanical Garden of Siberian Branch of the Russian Academy of Sciences, Russia, Novosibirsk, Russia; Central Siberian Botanical Garden of Siberian Branch of the Russian Academy of Sciences, Russia, Novosibirsk, Russia; Central Siberian Botanical Garden of Siberian Branch of the Russian Academy of Sciences, Russia, Novosibirsk, Russia; Institute of Cytology and Genetics of Siberian Branch of the Russian Academy of Sciences, Novosibirsk, Russia; Institute of Cytology and Genetics of Siberian Branch of the Russian Academy of Sciences, Novosibirsk, Russia

**Keywords:** Elymus, taxonomy, allopolyploids, genome constitution, CAPS markers, Elymus, таксономия, аллополиплоиды, геномная конституция, CAPS-маркеры

## Abstract

The genus Elymus L., together with wheat, rye, and barley, belongs to the tribe Triticeae. Apart from its economic value, this tribe is characterized by abundance of polyploid taxa formed in the course of remote hybridization. Single-copy nuclear genes are convenient markers for identification of source genomes incorporated into polyploids. In the present work, a CAPS-marker is developed to distinguish basic St, H, and Y genomes comprising polyploid genomes of Asiatic species of the genus Elymus. The test is based on electrophoretic analysis of restriction patterns of a PCR-amplified fragment of the gene coding for beta-amylase. There are about 50 Elymus species in Russia, and most of them are supposed to possess one of three haplome combinations, StH, StY and StHY. Boreal StH-genomic species endemic for Russia are the least studied. On the basis of nucleotide sequences from public databases, TaqI restrictase was selected, as it produced patterns of restriction fragments specific for St, H, and Y haplomes easily recognizable in agarose gel. A sample of 68 accessions belonging to 32 species was analyzed. In 15 species, the earlier known genomic constitutions were confirmed, but in E. kamoji this assay failed to reveal the presence of H genome. This unusual H genome was suggested to originate from a different Hordeum species. In 16 species, genomic constitutions were identified for the first time. Fifteen accessions from Asian Russia possessed the genomic constitution StStHH, and E. amurensis, phylogenetically close to the StY-genomic species E. ciliaris, had the genomic constitution StStYY. It is inferred that the center of species diversity of the StH-genomic group is shifted to the north as compared to the center of origin of StY-genomic species, confined to China. Key words: Elymus; taxonomy; allopolyploids; genome constitution; CAPS markers.

## Abstract (ru)

Род Elymus L. наряду с пшеницей, рожью и ячменем принадлежит к трибе Triticeae. Помимо своего хозяйственного значения, эта триба характеризуется широким распространением аллополиплоидных таксонов, которые формируются в ходе межвидовой и межродовой гибридизации и последующих преобразований вовлеченных в гибридизацию диплоидных геномов. Для идентификации исходных геномов в составе полиплоидов удобны малокопийные ядерные гены, менее подверженные процессам реорганизации, чем повторенные некодирующие элементы. В настоящей работе разработан удобный CAPS-маркер для различения базисных геномов St, H, Y, входящих в состав азиатских видов рода Elymus, с помощью электрофоретического анализа фрагментов рестрикции ПЦР-амплифицированного участка гена, кодирующего β-амилазу. В России распространено около 50 видов Elymus предположительно трех гапломных комбинаций: StH, StY и StHY. Наименее изученными остаются бореальные StH-геномные виды – эндемики Российской Федерации. По результатам анализа ранее изученных разными авторами нуклеотидных последовательностей гена β-амилазы была отобрана эндонуклеаза рестрикции TaqI, которая имела различающиеся по положению сайты узнавания в составе вышеуказанного фрагмента из геномов St, H и Y. В результате расщепления ПЦР- продукта эндонуклеазой TaqI у каждого из исходных гапломов формировался специфичный паттерн фрагментов рестрикции, легко визуализируемый в агарозном геле. Проанализирована выборка из 68 образцов, принадлежащих 32 видам. У 15 видов была подтверждена ранее известная геномная конституция, у E. kamoji этот метод не позволил выявить присутствие генома H. Предполагается возможное происхождение данного варианта генома H от другого вида Hordeum. У 16 видов геномная конституция определена впервые. Показано, что большинство изученных видов бореальной группы видов из Сибири и Российского Дальнего Востока имеют геномную конституцию StStHH. Исключение составил E. amurensis, филогенетически близкий к StY-геномному виду E. ciliaris и также имеющий геномный состав StStYY. Сделан вывод, что основное видовое разнообразие StH-геномной группы находится севернее центра происхождения большинства StY-геномных видов рода.

## Introduction

The genus Elymus L. is the largest in the tribe Triticeae Dum. and, according to different estimates, counts from 150 to 200 species (Dewey, 1984; Barkworth, 2000). It is represented only by alloploid taxa with genome compositions including several basic genomes (haplomes) in different combinations. The genetic basis of the genus Elymus is formed by five haplomes descending from different genera of the tribe Triticeae: (St) Pseudoroegneria, (H) Hordeum, (P) Agropyron, (W) Astralopyrum, (Y) donor unknown. Genome constitution was proposed as a stable genetic criterion for taxonomic classification of Elymus species (Löve, 1984). Within a relatively short span of time, substantial changes occurred in the taxonomy of the tribe Triticeae on the basis of the genomic system of classification suggested by D.R. Dewey (1984). During the next 20 years, six genera were identified according to variants of genome constitution: Douglasdeweya C. Yen, J.L. Yang & B.R. Baum (PPStSt), Roegneria C. Koch (StStYY), Anthosachne Steudel (StStWWYY), Kengylia C. Yen & J.L. Yang (PPStStYY), Campeiostachys Drobow (HHStStYY), and Elymus L. (StStHH, StStStHH, StStHHHH).

However, departing from A. Löve’s principles, many botanists still attribute several genome combinations to the single genus Elymus s. l. With all this, genome constitutions are not yet determined in about 40 % of species (Okito et al., 2009). According to current evidence, 53 species of the genus Elymus subdivided into four sections occur in Russia (Tsvelyov, 2008; Tsvelyov, Probatova, 2010). Two of the sections, Elymus and Goulardia (Husn.) Tzvelev, contain species with different genomic constitutions, which obviously contradicts the phylogenetic principle of their formulation. We suppose that Russia is home to species with only three haplome combinations: StH, StY, and StHY (Agafonov et al., 2015). Boreal StH-genomic endemics of Russia are less studied. According to the taxonomic system based on the genome constitution, the Elymus species should be attributed to three genera: Elymus, Roegneria, and Campeiostachys. However, in our view, the division of the species inhabiting Russia into three genera is impractical due to the difficulties of morphologic identification of these genera. With all this, taxonomic classification within the genus based on genome constitutions is indispensable for the construction of a phylogenetically oriented taxonomy of the genus.

Earlier, Cleaved Amplified Polymorphic Sequences (CAPS) markers were used to distinguish individual genomes in representatives of the tribe Triticeae (Gostimsky et al., 2005; Li et al., 2007; Hu et al., 2014; Shavrukov, 2016). Some advantages of CAPS markers are their codominance, moderate sensitivity to the amount of genomic DNA, and relatively low cost.

We were first to use CAPS-markers to identify the genomic constitutions of species of the genus Elymus (Kobozeva et al., 2017). For this purpose, primers were designed based on the known sequences of the gene coding for β amylase (Mason- Gamer, 2013), which included 38 sequences of haplome St, 23 of haplome H, and 15 of haplome Y, belonging to 24 Elymus species. Of them, 14 species had the genomic composition StStHH; 9, StStYY; and 1, StStHHUkUk (Elytrigia repens). Variable positions were sought that would discriminate representatives of an individual genome from the other two. Special attention was paid to those genome-specific sequence variants that resulted in appearance/disappearance of recognition sites for restriction endonucleases. It was found that digestion of the PCR products with TaqI endonuclease resulted in the formation of genome-specific restriction patterns. In the present work, we apply CAPS analysis to a large sample of Elymus species from Asian Russia to reveal their genome constitutions unknown hitherto.

## Materials and methods

Plant material included 68 accessions of the species with known (Table 1) and unknown (Table 2) genome constitutions found in Russia. The species nomenclature is given according to N.N. Tsvelyov and N.S. Probatova (2010). The accessions analyzed were received from the scientific collection of biological resources of the Central Siberian Botanic Garden SB RAS “Collections of living plants indoors and outdoors”; their identification numbers are given in Tables 1 and 2. Prefixes correspond to the geographic origin of the accessions.

**Table 1.**
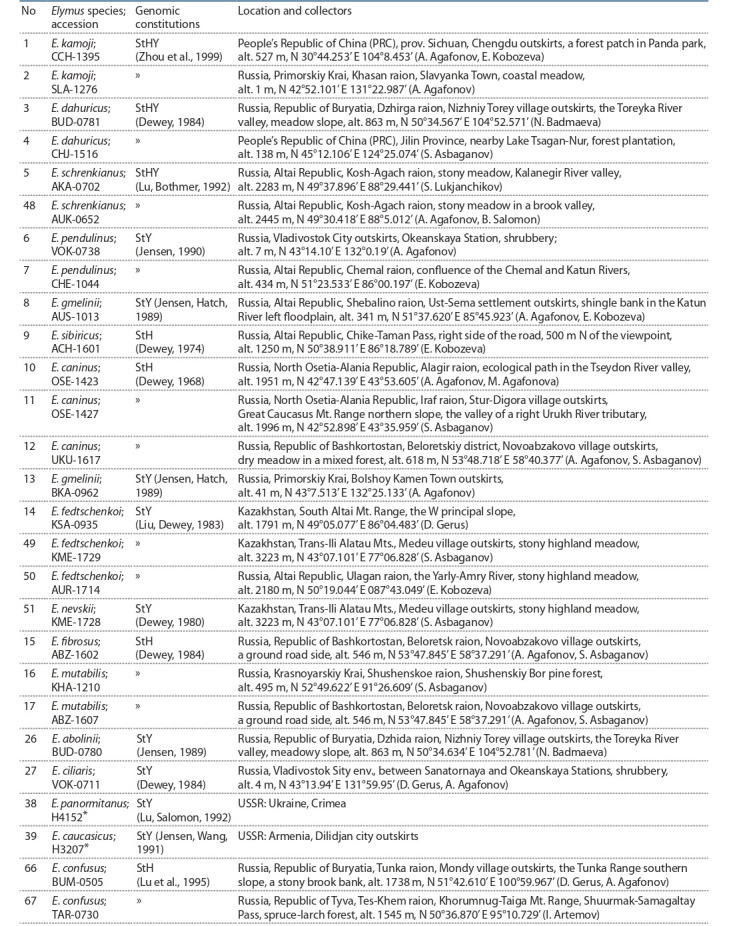
Accessions of Elymus species with known genomic constitutions determined by the classical cytogenetic method Note. The numbering of accessions corresponds to the lane numbering in Fig. 2. * Accessions kindly provided by Dr. B. Salomon (Swedish University of Agricultural Sciences, Department of Plant Breeding, Alnarp, Sweden).

**Table 2.**
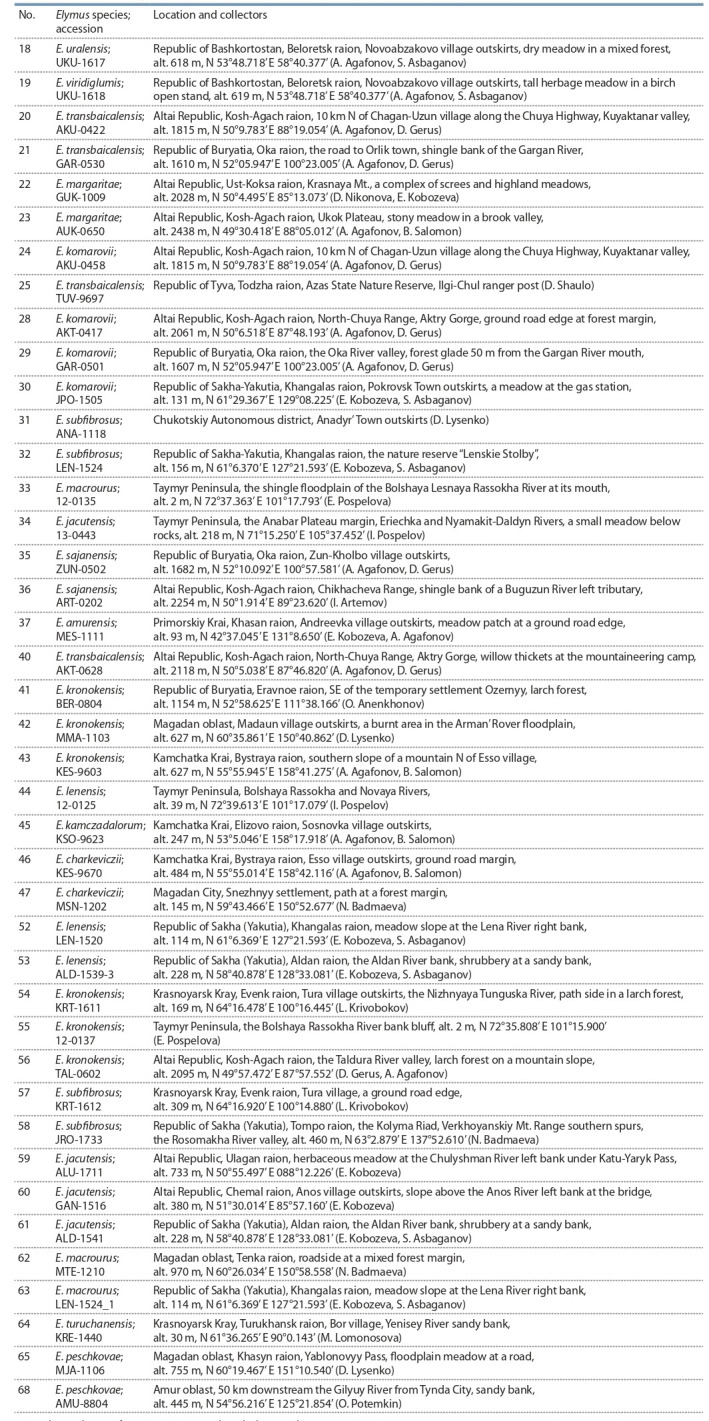
Accessions of Elymus species with unknown genomic constitutions collected in Russia Note. The numbering of accessions corresponds to the lane numbering in Fig. 2.

Total DNA was extracted from 20 mg of dried green matter with the use of NucleoSpin Plant II Kit (Macherey-Nagel, Germany) according to manufacturer’s recommendations. Amplification of the β amylase gene fragment was made in a C-1000 thermocycler (Bio-Rad, USA) with the following primers: El_balg_F4 (5ʹ-GGTACCATCGTGGACATTGAA- 3ʹ) and El_balg_R4 (5ʹ-CTGTACCACCAGTGAATGCC-3ʹ) (Kobozeva et al., 2017). The PCR reaction mixture of 15 μL in volume contained 1× buffer for Taq polymerase, 0.2 mM each dNTP, 1.5 mM MgCl_2_, 1 μM each of primers, 20 ng of genomic DNA, and 1 U of HS Taq DNA polymerase (Eurogene, RF). The following settings were used: predenaturation at 94 °С for 4 min; 40 cycles: denaturation at 94 °С for 20 s, primer annealing at 60 °C for 25 s, elongation at 72 °С for 90 s; postextension at 72 °С for 5 minutes. CAPS-analysis (Konieczny, Ausubel, 1993) was made as follows: 8 μL of the PCR reaction mixture was mixed with MQ-H_2_O and TaqI buffer up to 1× concentration in a volume of 15 μL, and 1 unit of TaqI restrictase (Thermo Scientific, USA) was added. The mixture was incubated at 65 °С for 1 hour and resolved in 1.7 % agarose gel in ТАЕ buffer. Molecular weight marker: 100+ bp DNA Ladder (Evrogen, RF).

## Results and discussion

**Fig. 1.**
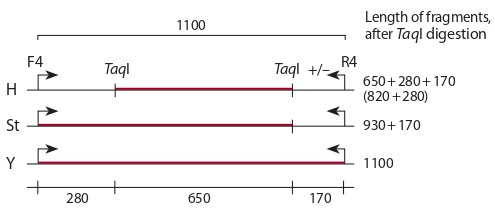
Map of recognition sites for TaqI endonuclease in the β amylase gene fragment amplified from the basic haplomes constituting the polyploid Elymus genome.

The comparative analysis of sequences of the β amylase gene published in R. Mason-Gamer (2013) showed that the studied fragment of Y genome of about 1100 bp in length did not contain recognition sites for TaqI endonuclease, while St genome contained one recognition site in the fragment of interest at a distance of about 170 bp from the primer El_balg_R4. The same site was present in some H genomes; besides, all H genomes contained a recognition site at a distance of about 280 bp from the primer El_balg_F4. Visualized on gels, restriction patterns of the studied genomes were differentiated according to the lengths of the longest fragments: Н genome was distinguished by the presence of a band at about 650 bp; St genome, 930 bp; and Y genome, 1100 bp (Fig. 1).

**Fig. 2.**
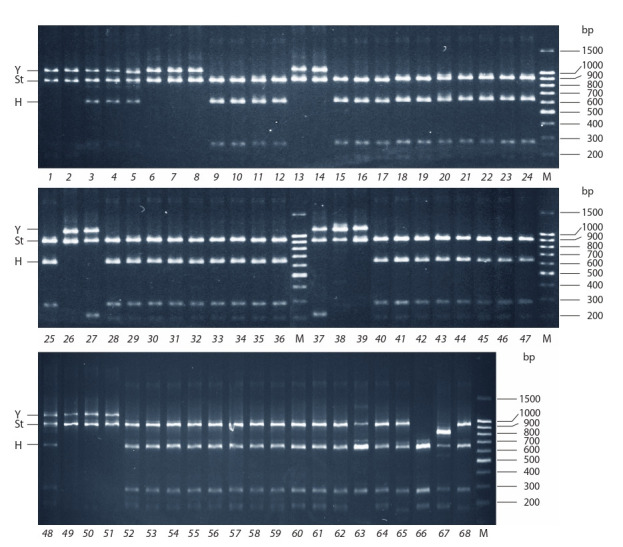
Polymorphism of restriction fragment lengths (CAPS) after TaqI digestion of the PCR-amplified fragment of the β amylase gene in species of the genus Elymus. Lane numbers correspond to the accession numbering in Tables 1 and 2. M – molecular weight ladder: 100+bp DNA Ladder (Evrogen).

Restriction patterns of the CAPS marker employed were studied in 68 accessions (see Tables 1, 2). Electrophoretic patterns formed after TaqI digestion are shown in Fig. 2. Based on the results of CAPS analysis, genomic constitutions of the accessions studied were determined. Previously known genomic constitutions were confirmed in 15 species of 16, E. kamoji being the only exception. In 16 species, genomic compositions were determined for the first time: 15 of them had the genomic constitution StStHH, and one species, E. amurensis, had StStYY (Table 3). However, some limitations of the approach were met. For example, in two accessions of E. kamoji CAPS-analysis revealed only two haplomes, St and Y (Fig. 2, lanes 1 and 2), whereas it is known to be hexaploid according to the number of chromosomes, thus, it should contain three basic genomes (haplomes). It is improbable that the absence of restriction fragments corresponding to haplome H was due to incomplete digestion. Since all representatives of the genus contain St haplome, possessing a recognition site for TaqI endonuclease, the presence of St-specific fragments serves as an internal control for the completeness of hydrolysis. According to the classification system based on genomic compositions, E. kamoji belongs to the genus Campeiostachys (Baum et al., 2011) which embraces species with the genomic composition StHY. In fact, we performed a cytological analysis, which showed that both accessions of E. kamoji possessed the chromosome number 2n = 42, corresponding to hexaploid. The presence of the H genome lacking two recognition sites for TaqI endonuclease in E. kamoji brings its origin into a question. It is not inconceivable that different representatives of the genus received their H genomes from different ancestor species, which agrees with the assumption of polyphyly of the donors of basic haplomes (Mason-Gamer, 2013).

**Table 3.**
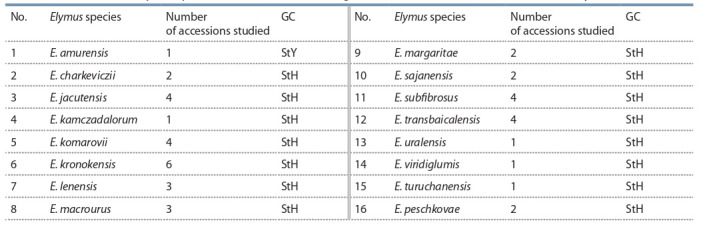
The list of boreal Elymus species in Asian Russia in which genome constitutions (GС) were determined by the CAPS method

An interesting pattern of restriction fragments was observed in two accessions of E. confusus (see Fig. 2, lanes 66 and 67), with the genome constitution formerly determined as StStHH (Lu et al., 1995). In accession TAR-0730 (see Fig. 2, lane 67), the longer fragment corresponding to the allele from St genome is truncated, possibly, as the result of a deletion or acquisition of an additional restriction site. The spectrum of restriction fragments in accession BUM-0505 (see Fig. 2, lane 66) lacks the fragment of about 930 bp characteristic of St genome, while the smaller fragment of about 170 bp corresponding to this haplome is clearly seen. This phenomenon might be attributed to a mutation in the St genome of the accession, for example, appearance of a recognition site for TaqI. Another possibility is a recombination and/or introgression between genomes St and H in the course of intense microevolutionary processes indirectly confirmed by the high morphologic variability within this species.

According to the CAPS analysis undertaken in the present work, almost all newly studied accessions of the boreal group of species from Siberia and Russian Far East have the StH genomic composition. One exception was E. amurensis, phylogenetically close to the StY-genomic species E. ciliaris and possessing the genome composition StY. This implies that the center of species diversity of the Asiatic StH-genome group is shifted to the north as compared to that of the StYgenome group, which is considered to be situated in China (Lu, Salomon, 1992). In this context, it is worth noting that in North America, the genus Elymus is also represented mainly by StH-genome species (except for Elymus californicus with unclear origin) (Mason-Gamer, 2001). Besides, in that territory a number of adventive Asiatic StHY- and StY-genome species were found (Barkworth et al., 2007).

In general, the applied method showed a high accuracy: in the present work earlier known genome constitutions were confirmed by CAPS analysis in 15 Elymus species of 16. For 10 species, the genomic composition newly determined by CAPS analysis as StH, was independently corroborated by sequencing of a cloned fragment of the GBSS1 (waxy) gene (Kobozeva et al., 2018; Agafonov et al., 2019). It should be noted that the sequencing of DNA from polyploid species has a disadvantage, as it is rather laborious, requiring additional gene cloning manipulations.

## Заключение

The main advantage of CAPS markers is the ease of their methodic implementation, which permits one to analyze many specimens with extensive morphologic and genetic variability from broad ranges. The present work involves CAPS analysis with the use of a fragment of the gene for β amylase and demonstrates rather good predictive power of the method. However, it should be kept in mind that no molecular marker taken by itself can unambiguously identify a genome or species; it serves as a marker, not diagnostic. Therefore, the development of additional simple and accessible approaches for genome identification in new and poorly studied biotypes from local habitats remains vital.

## Conflict of interest

The authors declare no conflict of interest.
